# Glomus tumour: an institutional experience of 31 cases

**DOI:** 10.1186/s13018-023-04234-1

**Published:** 2023-09-30

**Authors:** Wen Qiang Lee, Yihan Li, Nicholas Eng Meng Yeo

**Affiliations:** 1 Department of Orthopaedics, Singapore General Hospital, Outram Road, Singapore, 169608 Singapore; 2 Department of Anatomical Pathology, Singapore General Hospital, Outram Road, Singapore, 169608 Singapore

**Keywords:** Case series, Clinical features, Glomus tumour, Histological features, Uncommon soft tissue tumour

## Abstract

**Background:**

Glomus tumour is an uncommon soft tissue tumour which commonly occurs in the distal extremities, particularly the subungual region of the finger. Due to its rarity, there is a paucity of literature concerning glomus tumour. Therefore, this paper aims to report a case series based on our institution’s experience.

**Methods:**

A retrospective cross sectional study was performed in a single tertiary institution in Singapore. All patients diagnosed with glomus tumour confirmed on histology from January 2019 to October 2022 were included in the study. Patient demographics and clinical information (presenting signs and symptoms, tumour parameters and presence of recurrence) were retrieved from existing medical records.

**Results:**

A total of 31 cases of glomus tumour were diagnosed from January 2019 to October 2022, and the relevant demographics and clinical presentation were reported. Majority of glomus tumours occurred in the finger (61.3%). Pain was present in almost all the cases (96.8%), while a lump was visible in less than half (48.4%). An average of 44.0 months elapsed before patients were properly diagnosed and treated. There were no cases of recurrence despite involved margins in three cases.

**Conclusion:**

Glomus tumour can be easily missed if clinicians do not have an index of suspicion for it, resulting in delayed treatment. Once diagnosed, glomus tumour can be treated with complete excision with good outcomes.

## Background

Glomus tumours are uncommon neoplasms arising from the glomus body, which is part of the thermoregulation pathway in the dermis [1, 2]. First described in 1812 by Wood [3] to be a painful subcutaneous tubercle, glomus tumours are commonly found in the digits of the upper extremities [1, 2]. However, since then, there have been case reports of glomus tumours in other locations, including viscera such as the eye [4], lung [5], stomach [1] and kidney [6].

The classical symptoms of glomus tumour, as described by Carroll et al. [7], are pain, tenderness and cold sensitivity. Signs on physical examination include Love test (point tenderness) and Hildreth’s sign (reduction in pain/tenderness after a tourniquet is inflated above systolic blood pressure proximal to the tumour) [8, 9]. While a history of preceding trauma is sometimes associated with this neoplasm, no definite causative relationship has been established [10].

**Fig. 1:**
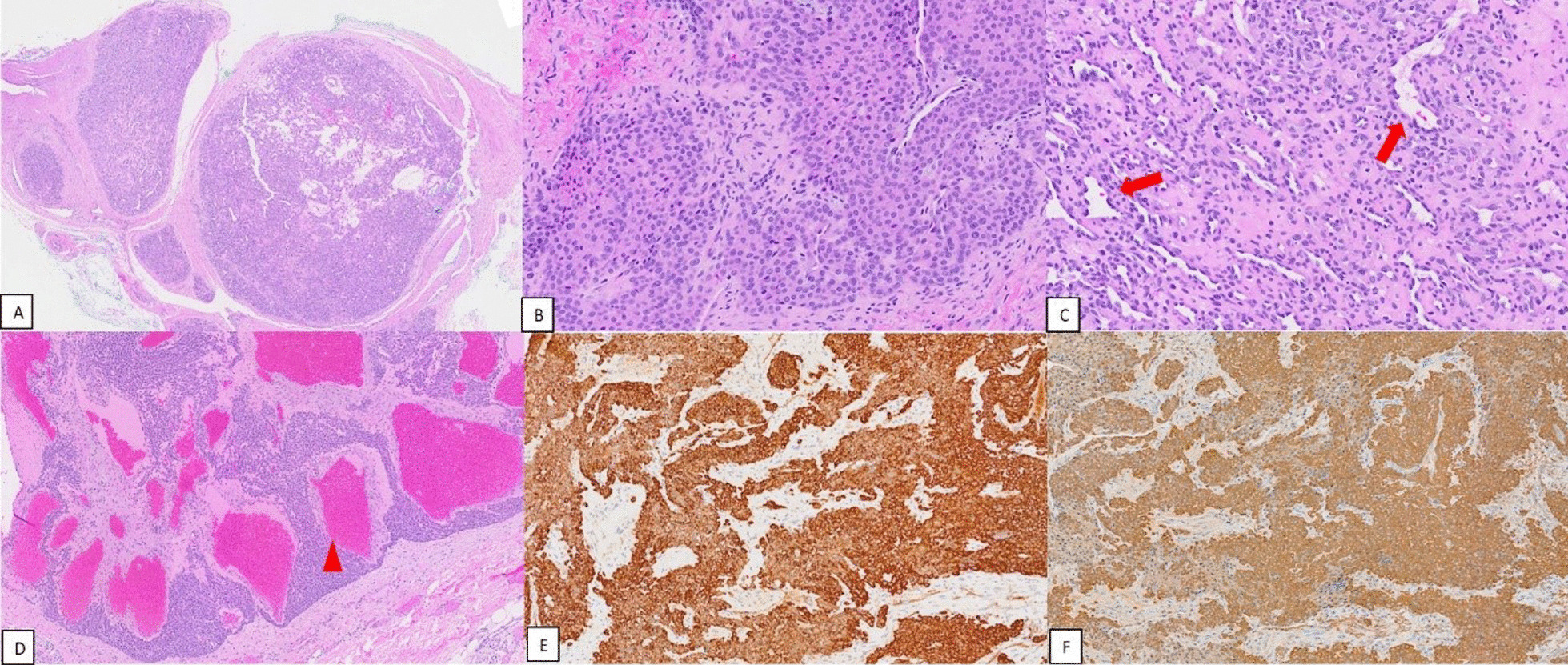
Histopathological slides of glomus tumour: **A** Low-power view demonstrating a well-circumscribed nodule (H&E, × 2 magnification); **B** Glomus tumour cells are small, round to ovoid, uniform cells, with pale to light eosinophilic cytoplasm, centrally located nuclei and sharply defined cell borders (H&E, × 20 magnification); **C** Glomus cells surrounding capillary-sized vessels (red arrows) (H&E, × 10 magnification); **D** Glomus cells surrounding cavernous hemangioma-like vascular structures (red arrowhead), known as glomangioma (H&E, × 5 magnification); **E** and **F** Glomus cells stain diffusely for caldesmon and smooth muscle actin (**E** Caldesmon, × 20 magnification), **F** SMA, × 20 magnification))

Histologically, glomus tumours are usually well-circumscribed nodules comprised of uniform round cells with centrally located nuclei and well-defined cell borders (Fig. 1A, B). The cells are generally peri-vascular in arrangement (Fig. 1C). Glomangiomas, a common variant of glomus tumour, tend to have a more prominent vascular component (Fig. 1D). Glomus tumours tend to stain strongly for smooth muscle actin (SMA) and caldesmon (Fig. 1E, F) [11].

Generally considered to be a benign neoplasm, there have been cases of malignant transformation [12, 13]. Some of the features suggestive of malignant potential are deep location, large size > 2 cm, atypical mitotic figures, high nuclear grade and high mitotic activity > 5 mitoses/50 high power field [13].

Despite being described more than a century ago, most of the available literature on glomus tumours is limited to case reports and a few case series. In this study, we present a series of 31 cases of glomus tumours in our institution, including a case report of a subungual glomus tumour of the toe, which to the authors’ knowledge, is one of the larger studies available. In doing so, we hope to further shed light on the clinical characteristics of this uncommon neoplasm.

## Materials and methods

A retrospective cross sectional study was performed in a single tertiary institution in Singapore. All patients diagnosed with glomus tumour confirmed on histology by independent pathologists in our institution from January 2019 to October 2022 were included in the study.

The cases were identified through our institution’s histopathology record database. Relevant clinical information of included patients was retrieved from existing medical records. The following information was collected: Demographics (age, gender, and race), presenting signs and symptoms (duration of symptoms, pain, presence of lump, discoloration, and presence of preceding trauma), tumour parameters based on surgical or histological reports (location, size, and margins) and presence of recurrence on last clinical follow-up.

Continuous variables were presented as mean and standard deviation, while categorical variables were presented in terms of frequency and percentage of the whole sample.

This study was approved by our institution’s ethics committee (Centralized Institutional Review Board Reference Number: 2022/2475).

## Results

A total of 31 cases were included in the study. The mean age of presentation was 48.8 years, and there was equal distribution between genders. Most had a histological diagnosis of glomus tumour (93.5%), with only two cases diagnosed as glomangioma.

Majority of glomus tumours was found in the extremities, with the fingers being the most common location (61.3%), followed by around the knee region (16.1%). One case had a glomus tumour located in the left 11th rib, while another was found in the stomach. Pain was the defining presenting symptom in almost all cases (96.8%), except for the tumour located in the stomach. A visible lump was only present in less than half of the cases (48.4%). The presence of preceding trauma was found in only 16.1% of the cases in this study. Symptoms were present for an average of 44.0 months before initial consultation. Table 1 shows the characteristics of the glomus tumours.

**Table 1:** Characteristics of glomus tumour

Parameters	Number (*n* = 31)	Percentage (%)
Age (years)	48.8 ± 14.0	–
*Gender*		
Male	16	51.6
Female	15	48.4
Race		
Chinese	22	71.0
Malay	4	12.9
Indian	4	12.9
Others	1	3.2
*Type of tumour*		
Glomus	29	93.5
Glomangioma	2	6.5
Duration of symptoms (months)	44.0 (± 38.3)	–
Pain	30	96.8
Swelling/lump	15	48.4
Discoloration	7	22.6
Presence of preceding trauma	5	16.1
*Location of tumour*		
Toe	1	3.2
Finger	19	61.3
Knee	5	16.1
Others^*^	6	19.4
Size (mm^2^)	626 (± 2018)	–
Clear margins on histology	29	93.5
Duration of follow-up (days)	174 (± 267)	–
Recurrence	0	0

^*^ Others: 2 cases in the hand, 1 case in the wrist, 1 case in the forearm, 1 case in the stomach^1^ and 1 case in the rib

^1^The patient was undergoing investigation for newly diagnosed metastatic rectal carcinoma when a 1.8 × 1.8 × 1.5 cm tumour was incidentally picked up on the CT thorax, abdomen and pelvis scan, with a provisional diagnosis of gastrointestinal stromal tumour. A wedge resection of the stomach was performed during the anterior resection surgery, and the stomach tumour was found to be a glomus tumour

All the patients underwent an excisional biopsy, of which two were reported to have involved margins. The two cases with involved margins are as follows: Glomus tumour of the right knee followed-up for 785 days and glomus tumour of the 11th rib followed-up for 638 days. Overall, after an average follow-up period of 174 days across the 31 cases, there was no case of recurrence. All the cases were histologically benign, except for the 11th rib glomus tumour which had atypical features (deep location, 4.5 cm in largest diameter and high mitotic rate 10–15 mitoses/50 high power field). No adjuvant treatment was performed.

## Discussion

Glomus tumour is often encountered by hand or orthopaedic surgeons, owing to its usual occurrence in the extremities, especially the digits of the upper extremities. Majority of the cases in this study were found in the fingertips, which is similar to current literature [2, 8], where subungual glomus tumours of the finger account for up to 75% of cases. In our study, even those found in other locations tend to be within the extremities, such as the knee, toe, hand and wrist. This is concordant with a series of 56 extra-digital glomus tumours reported by Schiefer et al. [14], where 91% were in the extremities.

Glomus tumours are reported to occur more commonly in women, and around the age of 20 to 40 years old [2, 8]. Interestingly, in our study, there was an equal distribution between the genders. This could be due to the inclusion of glomus tumours outside the subungual region, which has been shown to have greater predilection in men [9, 14].

**Fig. 2:**
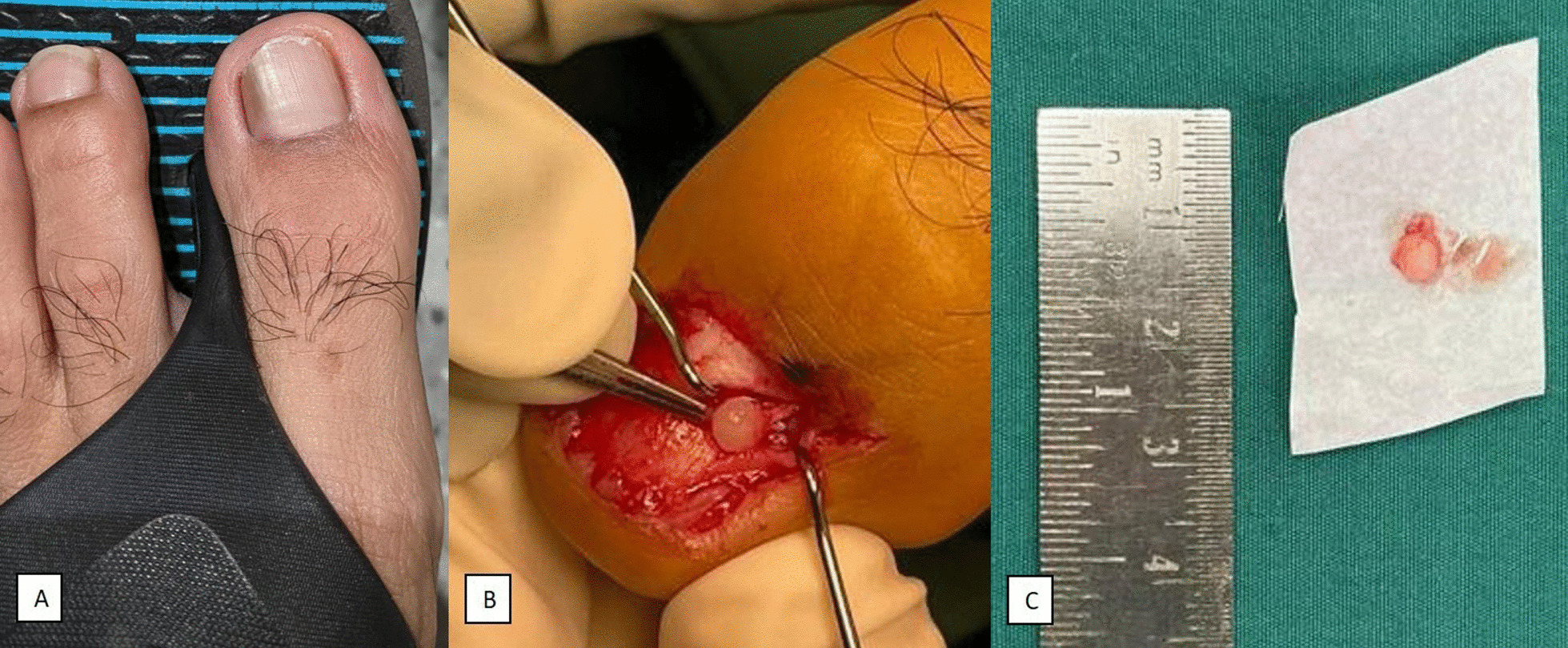
**A** Clinical photo showing slight discoloration of the lateral aspect of the 1st toenail **B** Clinical photo showing glomus tumour being enucleated from patient’s 1st toe nailbed. **C** Glomus tumour in (B) measured to be approximately 5 × 3 mm

**Fig. 3:**
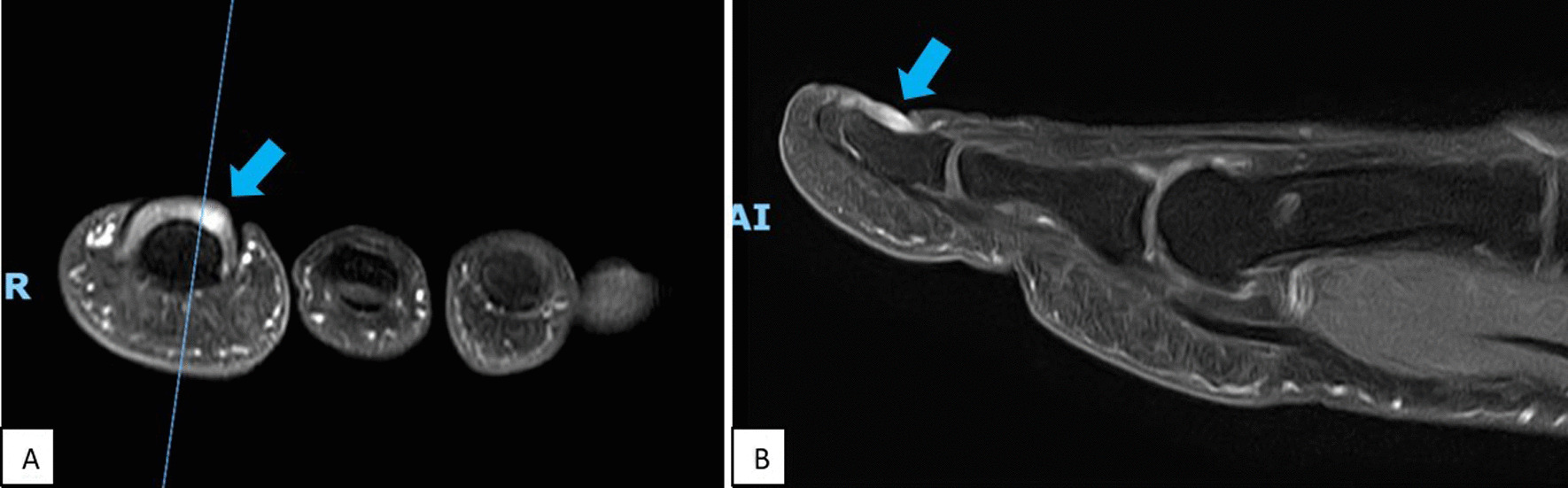
T2-weighted coronal **A** and sagittal **B** cuts of the patient’s left big toe. Contrast-enhancing nodule (blue arrows) noted in the proximal lateral subungual region with no obvious cortical erosion of the distal phalanx

The clinical presentation of the patients in our study is comparable with that of the literature [8, 15], with most presenting with highly localised pain. Of note, less than half of the patients had a clinically apparent lump and even fewer had skin changes/discoloration. This could explain the relatively long average duration of almost four years before the diagnosis of glomus tumour was made in our case series. In fact, delayed diagnosis is often described in the literature [1, 2, 10, 12]. To highlight a case example in this study, a patient presented with left big toe pain and slight bluish discoloration of the lateral aspect of his toenail (Fig. 2) with exquisite point tenderness over the area. However, he was treated for other conditions such as ingrown toenail and onychomycosis before the correct diagnosis was clinched after a magnetic resonance imaging (MRI) scan (Fig. 3) was performed. Therefore, clinicians should consider the diagnosis of glomus tumour should they encounter patients with similar presentation.

Radiological investigations may be used to supplement clinical suspicion. Although a soft tissue tumour, glomus tumour may sometimes result in scalloping of adjacent bone which can be picked up on plain radiographs [2, 11, 14]. Ultrasound Doppler has been used to diagnose glomus tumour as well, owing to its hypervascular nature [2, 11]. However, MRI scan is one of the more commonly used modalities to diagnose glomus tumour, which classically appears as a well-circumscribed nodule that is hypointense on T1-weighted images and hyperintense on T2-weighted images [2, 11, 14].

Complete excision is considered curative, often with good outcomes [14]. Almost all the cases in our study had benign histological diagnosis, and those with involved resection margins did not suffer recurrence during their follow-up. In general, most studies in the literature also report a low rate of recurrence [16, 17], although some report a recurrence rate of up to 30% [9]. In a case series of 32 patients with glomus tumour considered to be malignant [13], only 2 of 15 patients with involved resection margins had recurrence. However, the authors acknowledge that the average follow-up duration in this study is relatively short at approximately 5.8 months, and further studies with longer follow-up duration would provide a clearer picture on the recurrence rate and clinical outcomes.

The key strength of this study is the number of patients, representing one of the largest in the current literature. The clinical characteristics and outcomes presented will hopefully shed further light on this condition and aid clinicians in managing patients with this uncommon neoplasm.

## Conclusion

Glomus tumour is an uncommon soft tissue neoplasm that commonly occurs in the extremities. As it is often clinically unapparent aside from the presence of significant pain, it may be easily dismissed by clinicians, resulting in a prolonged period before the right diagnosis is made. While it is usually benign with a seemingly low recurrence rate, clinicians should have a high index of suspicion for those which have atypical features.

## Data Availability

The datasets used and/or analysed during the current study are available from the corresponding author on reasonable request.

## References

[1] Trehan SK, Soukup DS, Mintz DN, Perino G, Ellis SJ (2015). Glomus tumors in the foot. Foot Ankle Spec.

[2] Romanos E, Al Delfi F, Hubballah M, Farah C (2019). Glomus tumour of the fourth toe: Case discussion and review of literature. BMJ Case Reports.

[3] Wood W (1812). On painful subcutaneous tubercle. Edinb Med Surg J.

[4] Jing H, Weiwen C, Meihong C, Xiaohong G (2020). Glomus tumour of the iris: a case report. Eur J Ophthalmol.

[5] Singh V, Kumar V, Singh H, Kakkar N (2020). Primary pulmonary glomus tumour: A diagnostic challenge. BMJ Case Reports..

[6] Novis E, Raman A, Maclean F, Lazzaro E (2016). Glomus tumour of the kidney: A case report and review of the literature. ANZ J Surg.

[7] Roberte C, Arnoldt B (1972). Glomus tumors of the hand. J Bone Joint Surg.

[8] Sethu C, Sethu AU (2016). Glomus tumour. Annals Royal College Surg England..

[9] Polo C, Borda D, Poggio D, Asunción J, Peidro L (2012). Glomus tumor of the hallux review of the literature and report of two cases. Foot Ankle Surg.

[10] Mohindra M, Sambandam B, Gautam VK, Maini L (2016). A rare case of glomus tumor of the Great Toe. Foot Ankle Spec.

[11] Mravic M, LaChaud G, Nguyen A, Scott MA, Dry SM, James AW (2015). Clinical and histopathological diagnosis of glomus tumor. Int J Surg Pathol.

[12] Sacchetti F, De Gori M, Grossi S, Bonadio GA, Capanna R (2019). An exceptional case of malignant glomus tumor and a review of the literature. Acta Orthop Traumatol Turc.

[13] Folpe AL, Fanburg-Smith JC, Miettinen M, Weiss SW (2001). Atypical and malignant glomus tumors. Am J Surg Pathol.

[14] Schiefer TK, Parker WL, Anakwenze OA, Amadio PC, Inwards CY, Spinner RJ (2006). Extradigital glomus tumors: A 20-Year experience. Mayo Clin Proc.

[15] Van Geertruyden J, Lorea P, Goldschmidt D, de Fontaine S, Schuind F (1996). Glomus tumours of the hand. Journal of Hand Surgery.

[16] Lee W, Kwon SB, Cho SH, Eo SR, Kwon C (2015). Glomus tumor of the hand. Arch Plast Surg.

[17] Tomak Y, Akcay I, Dabak N, Eroglu L (2003). Subungual glomus tumours of the hand: Diagnosis and treatment of 14 cases. Scand J Plast Reconstr Surg Hand Surg.

